# Association of frailty with cognitive impairment and functional disability in older adults with affective disorders: a brief research report

**DOI:** 10.3389/fpsyt.2023.1181997

**Published:** 2023-07-11

**Authors:** Ariane M. Monteiro, Marcus K. Borges

**Affiliations:** ^1^Department of Internal Medicine, Post-Graduate Program of Internal Medicine and Health Sciences (PPGMICS), Complexo do Hospital de Clínicas, Universidade Federal do Paraná (CHC-UFPR), Curitiba, Brazil; ^2^Department of Internal Medicine and Psychiatry (Geriatric Psychiatry), Post-Graduate Program of Internal Medicine and Health Sciences (PPGMICS), Complexo do Hospital de Clínicas, Universidade Federal do Paraná (CHC-UFPR), Curitiba, Brazil

**Keywords:** older adults, IVCF-20, frailty, cognitive impairment, functional disability

## Abstract

**Introduction:**

The Clinical-Functional Vulnerability Index (IVCF-20) is a validated multidimensional instrument that has been used in Brazil to evaluate functional disability in frail older adults. The main aim of this study was to assess frailty using this novel screening tool. In addition, to investigate whether frailty was associated with cognitive impairment and functional disability in older adults with affective disorders.

**Methods:**

Participants included were over 60 years old, with affective disorders (depressive or anxiety disorders), from two specialized outpatient clinics. The sample was comprised of 46 patients (30% of a total from 153). The following instruments were applied: Clock Drawing Test (CDT), Mini Mental State Examination (MMSE); Verbal Fluency Test (VFT); Pfeffer Questionnaire or Functional Assessment Questionnaire (FAQ); Katz Index; Geriatric Depression Scale (GDS-15); Geriatric Anxiety Inventory (GAI), and IVCF-20 as well as sociodemographic and clinical questionnaires. The association between the variables of interest was estimated using Spearman correlation.

**Results:**

This study found a negative correlation between frailty and cognitive decline (MMSE; rs = −0.58; *p* < 0.001); (VFT; rs = −0.60; *p* < 0.001); (CDT; rs = −0.47; *p* = 0.001) and a positive correlation between frailty and depressive symptoms (GDS-15; rs = 0.34; *p* = 0.019) as well as disability for IADLs (FAQ; rs = 0.69; *p* < 0.001). However, there was no statistical difference in the association between frailty and anxiety symptoms (GAI; rs = 0.24; *p* = 0.103) or disability for BADLs (Katz; rs = −0.02; *p* = 0.895).

**Discussion:**

Our data support that the associations between frailty, cognitive and functional disability are prevalent issues in Psychogeriatrics. Assessing frailty in a multidimensional context is essential using a rapid assessment frailty tool in clinical practice.

## 1. Introduction

According to the World Health Organization (1) populations around the world are experiencing a healthy life expectancy in a context of rapid global aging. Unfortunately, a higher life expectancy leads to burden of diseases and disability in older people in the Americas (2). Recent studies have addressed frailty, a condition related to a loss of homeostasis and a deregulation of multiple systems as well as physiological reserve against different types of stressors (3). This reduced ability to respond to stress is due to the multiple deficits accumulated with biological aging, which generates this loss of homeostasis (4). The early detection of this health condition is essential, in order to reverse or reduce cognitive and functional decline, providing a better quality of life in the context of healthy aging.

Frailty, cognitive impairment and affective disorders are prevalent issues in Psychogeriatrics (5). Affective disorders are associated with a poor quality of life in the elderly and often coexisting with frailty (6). The most common mental disorders among older adults are major depression (MD) and generalized anxiety disorder (GAD) (7). Depressive symptoms increase the risk of cognitive impairment and functional disability (8). In addition, late-life depression (LLD) is frequently associated with cognitive decline (9) and geriatric anxiety also leads to changes in the neuropsychological functioning (10). Therefore, it has been proved that there is a reciprocal relationship between affective as well as cognitive disorders and frailty in older adults (6, 11).

There is still a gap in research investigating frailty in the context of geriatric psychiatry (12). This study contributes to the existing body of knowledge by investigating the phenomenon of frailty in geriatric patients affected by pre-existing psychiatric disorders, thereby providing supplementary insights into the subject matter. A systematic review found a total of 51 frailty assessment instruments that were analyzed in 96 studies published between 1997 and 2018 (13). There were 9 studies published in Portuguese for use in Brazil. A total of 9 instruments, such as: Frailty Phenotype Modified; FRAIL Scale; Edmonton Frail Scale (EFS); Gronigen Frailty Indicator (GFI); Tilburg Frailty Indicator (TFI); Instrumento Multidimensional de rastreio da Síndrome da Fragilidade (IMSIFI); Índice de Vulnerabilidade Clínico-Funcional (IVCF-20); Kihon Check-List (KCL); and PRISMA-7; have assessed physical and psychological domains. The FRAIL Scale, EFS, IVCF-20, GFI and TFI instruments were the most frequently analyzed in relation to clinimetric properties (13).

Although there are many studies about assessment frailty tools (13), a few studies focus on a validated multidimensional instrument that has been used in Brazil and evaluates disability in frail older adults. This novel instrument called IVCF-20 (14) consisting of 20 items, and 15 domains that are considered predictors of functional disability in the elderly, such as: age; self-perception of health; activities of daily living—ADLs; cognition; mood/behavior; mobility (reach, grip; aerobic/muscular capacity; gait); communication (vision and hearing); and comorbidities (multimorbidity, polypharmacy and/or recent hospitalization). It is quick to administer (5 to 10 min) and has the advantage of being available as a mobile application (app), which facilitates the data collection and minimizes potential measurement and recall biases (14). The aim of this study was to assess frailty using the IVCF-20, as well as, to investigate whether frailty is associated with cognitive impairment and functional disability in older adults with affective disorders.

## 2. Methods

### 2.1. Design, setting, and participants

We performed a cross-sectional study based on collected data in baseline. The study population was recruited from two specialized Psychogeriatric outpatient clinics in the south of Brazil. The data was collected from January 2022 to December 2022 and a sample of 46 patients (30% of a total from 153) was selected for this preliminary analysis including participants over 60 years old with affective disorders (depressive or anxiety disorders) referred these clinics. The assessments were conducted subsequent to the initial consultation at the clinic, during which the patients demonstrated manifestations of psychiatric symptomatology (anxious and/or depressive symptoms) and showed cognitive complaints. Both participants and caregivers have accepted to participate in the research and have signed the Informed Consent Form (ICF). Eligible patients were clinically evaluated for the inclusion criteria of a depressive or anxiety disorder and exclusion of another mental disorder as well as a depressive syndrome secondary to a somatic or clinical condition by two geriatric psychiatry specialists. The exclusion criteria were: (1) lost to follow-up in the clinics; (2) refusal to participate in the research; (3) refusal of the caregiver to participate in the research; (4) psychotic disorder or schizophrenia; (5) delirium or hospitalization in the last month; (6) previous electroconvulsive therapy (ECT); (7) severe sensory impairment; (8) unstable medical condition (e.g., decompensated heart failure, current infection); (9) terminal illness; (10) bipolar disorder; (11) post-traumatic stress disorder (PTSD); and (12) dementia. This study was approved by the Medical Research Ethical Committee from CHC-UFPR (CAAE number: 6556222.0.0000.0096).

### 2.2. Measurements

The assessment was performed in a single interview and included a sociodemographic and clinical questionnaire; frailty assessment (primary outcome) using the IVCF-20 (14); cognitive assessment (secondary outcome) using the Clock Drawing Test (CDT) (15), Mini Mental State Examination (MMSE) (16) and Verbal Fluency Test (VFT) (17); functional assessment (secondary outcome) using Pfeffer Questionnaire or Functional Assessment Questionnaire (FAQ) (18) and Katz Index (19); and affective disorders assessment using Geriatric Depression Scale (GDS-15) (20) and Geriatric Anxiety Inventory (GAI) (21). The interviews lasted from 30 to 40 min including the participation of both the patient and the caregivers. The only tool answered by the caregiver was the FAQ.

### 2.3. Statistical analysis

Socio-demographic and clinical data were distributed in two groups among patients diagnosed as non-frail (IVCF < 15) and frail (IVCF ≥ 15). Both groups were compared using the Chi-square or Fisher tests, as well as, data from the other instruments assessed. Yates’ correction was applied for the chi-square test due to the small sample. Prevalence ratios (PR), as well as the 95% confidence interval (95%CI), were calculated in order to measure the risk of frailty (primary outcome). Analysis of normality was performed using the Shapiro–Wilk test, which indicated that the data were nonparametric (*p* > 0.05). Thus, continuous variables were compared between the two groups using the Mann–Whitney test. Spearman’s correlation was also applied to assess the correlation between variables of interest. A *p-*value < 0.05 was considered significant. All analyses were performed using SPSS v.23.0 software (IBM-SPSS, United States).

## 3. Results

A total of 46 participants have been preliminary analyzed (from a total of 153). The study found a frailty prevalence in the sample of 41.31% and a statistically significant difference between the two groups (non-frail/frail) in relation to the following variables: multimorbidity (PR = 5; *p* = 0.031), polypharmacy (PR = 4.11; *p* = 0.018), need for a caregiver (PR = 2.38; *p* = 0.036) and marital status (*p* = 0.043). Due to the PR ≥ 1, we can suppose that multimorbidity, polypharmacy and the need for a caregiver increase the risk of frailty in our sample (Table 1).

**Table 1:** Distribution of sociodemographic and clinical variables between 2 groups (Non-frail/frail).

		Non-frail		Frail		
		*N*	%	*N*	%	*p*-value	PR (95% CI)
Total		27	58.69%	19	41.31%	-	
Sex	Male	14	51.85%	4	21.05%	0.072	2.41 (0.95–6.11)
	Female	13	48.15%	15	78.95%		
Retirement	Yes	25	92.59%	19	100.00%	0.504^f^	-
	No	2	7.41%	0	0.00%		
Current alcohol use	No	23	85.19%	18	94.74%	0.387^f^	0.45 (0.07–2.72)
	Yes	4	14.81%	1	5.26%		
Current smoking	No	27	100.00%	18	94.74%	0.413^f^	-
	Yes	0	0.00%	1	5.26%		
Regular physical exercise	<3 times a week	19	70.37%	17	89.47%	0.160^f^	0.42 (0.11–1.53)
	≥3 times a week	8	29.63%	2	10.53%		
Multimorbidity	<2 diseases	9	33.33%	1	5.26%	0.031^*^^f^	5.00 (0.75–33.02)
	≥2 diseases	18	66.67%	18	94.74%		
Polypharmacy	<5 medications	13	48.15%	2	10.53%	0.018^*^	4.11 (1.08–15.53)
	≥5 medications	14	51.85%	17	89.47%		
Hospitalization during past 6 months	No	26	96.30%	18	94.74%	>0.999^f^	1.22 (0.29–5.11)
	Yes	1	3.70%	1	5.26%		
Caregiver need	No	26	96.30%	14	73.68%	0.036^*^^f^	2.38 (1.36–4.14)
	Yes	1	3.70%	5	26.32%		
Marital status	Married	21	77.78%	11	57.89%	0.043^*^	-
	Single	2	7.41%	0	0.00%		
	Widow(er)	1	3.70%	7	36.84%		
	Separated	1	3.70%	0	0.00%		
	Divorced	2	7.41%	1	5.26%		
Education	0–4 years	4	14.81%	6	31.58%	0.179	-
	5–8 years	2	7.41%	4	21.05%		
	9–11 years	7	25.93%	4	21.05%		
	≥12 years	14	51.85%	5	26.32%		
Color	White	24	88.89%	15	78.95%	0.226	-
	Brown	3	11.11%	2	10.53%		
	Black	0	0.00%	2	10.53%		

Socio-demographic and clinical data were distributed in two groups among patients diagnosed as non-frail (IVCF < 15) and frail (IVCF ≥ 15). Both groups were compared using the Chi-square or ^f^Fisher tests. Yates’ correction was applied for the Chi-square test due to the small sample. Prevalence ratios (PR), as well as the 95% confidence interval (95%CI), were calculated in order to measure the risk of frailty (primary outcome).

* *p*-value < 0.05 was considered significant.

According to Table 2, regarding the tools used to assess symptoms of depression and anxiety (GAI and GDS-15), as well as those for cognitive (MMSE, CDT, and VFT) and functional assessment (PFEFFEER and Katz), results show a statistically significant difference in the Clock Test (CDT; PR = 2.57; *p* = 0.014) and functional disability for instrumental activities of daily living—IADLs (PFEFFER; PR = 6.34; *p* < 0.001). Although there was no difference in the other tests, we can suppose that the risk for frailty increases in individuals with worse performance in the GDS-15 (PR = 1.64); GAI (PR = 1.52); and MMSE (PR = 2.84).

**Table 2:** Distribution of the tools according to cut-off used between 2 groups (Non-frail/frail).

		Non-frail		Frail		
		*N*	%	*N*	%	*p*-value	PR (95% CI)
Geriatric depression scale (GDS-15)	<6	10	37.04%	4	21.05%	0.404	1.64 (0.66–4.06)
	≥6	17	62.96%	15	78.95%		
Geriatric anxiety inventory (GAI)	<13	13	48.15%	6	31.58%	0.412	1.52 (0.70–3.28)
	≥13	14	51.85%	13	68.42%		
Clock drawing test (CDT)	Normal	22	81.48%	8	42.11%	0.014^*^	2.57 (1.30–5.08)
	Abnormal	5	18.52%	11	57.89%		
Mini mental state examination (MMSE)	No	13	48.15%	3	15.79%	0.051	2.84 (0.97–8.32)
	Yes	14	51.85%	16	84.21%		
Verbal fluency test (VFT)	Normal	21	77.78%	9	47.37%	0.069	2.08 (1.07–4.05)
	Abnormal	6	22.22%	10	52.63%		
Disability for IADLs (PFEFFER)	No	22	81.48%	3	15.79%	<0.001^*^	6.34 (2.13–18.84)
	Yes	5	18.52%	16	84.21%		
Disability for BADLs (KATZ)	No	27	100.00%	18	94.73%	.413^f^	-
	Yes	0	0.00%	1	5.27%		

Both groups were compared using the Chi-square or ^f^Fisher tests, as well as, data related to instruments assessed. Yates’ correction was applied for the Chi-square test due to the small sample. Prevalence ratios (PR), as well as the 95% confidence interval (95%CI), were calculated in order to measure the risk of frailty (primary outcome).

* *p*-value < 0.05 was considered significant.

Table 3 shows the comparison between continuous variables and the non-frail/frail groups. It shows that there is a statistically significant difference in the following tests: CDT (*p* < 0.001), MMSE (*p* = 0.005), VFT (*p* < 0.001), and PFEFFER (*p* < 0.001). Thus, the worse the performance in the CDT, in the MMSE, in the VFT and in the PFEFFER, the higher the probability of frailty. The same was not observed regarding affective symptoms (GAI and GDS-15), and disability for basic activities of daily living—BALDs (Katz), since there was no statistical difference.

**Table 3:** Comparison of the continuous variables between 2 groups (Non-frail/frail).

	Non-frail			Frail			
	Median	Lower quartile (median)	Upper quartile (median)	Median	Lower quartile (median)	Upper quartile (median)	*p*-value
AGE	69.00	66.00	74.00	72.00	66.00	77.00	0.254
GDS-15	6.00	5.00	8.00	8.00	6.00	11.00	0.066
GAI	13.00	12.00	16.00	13.00	10.00	18.00	0.319
IVCF-20	8.00	8.00	12.00	19.00	16.00	21.00	<0.001^*^
CDT	10.00	10.00	10.00	8.00	5.00	10.00	0.005^*^
MMSE	27.00	27.00	29.00	23.00	22.00	25.00	<0.001^*^
VFT	14.00	13.00	17.00	10.00	9.00	12.00	<0.001^*^
PFEFFER	3.00	3.00	5.00	9.00	6.00	12.00	<0.001^*^
KATZ	6.00	6.00	6.00	6.00	6.00	6.00	0.361

Analysis of normality was performed using the Shapiro–Wilk test, which indicated that the data were nonparametric (*p* > 0.05). Thus, continuous variables and Medians were compared between the two groups using the Mann–Whitney test.

* *p*-value < 0.05 was considered significant.

Table 4 correlates the variables of interest. Regarding the primary outcome assessed by the IVCF-20, it shows a positive correlation between frailty and depressive symptoms (GDS-15; rs = 0.344; *p* = 0.019); and functional disability for IADLs (PFEFFER; rs = 0.692; *p* = 0.015). There was no statistically significant correlation between anxiety symptoms (GAI) and cognitive impairment or functional disability for BADLs (KATZ). It has shown that there was a negative correlation between frailty and cognitive impairment [CDT (rs = −0.473; *p* = 0.001); MMSE (rs = −0.581; *p* < 0.001); and VFT (rs = −0.601; *p* < 0.001)]. Regarding secondary outcomes (cognitive impairment and functional disability), there was a positive correlation between TDR and MMSE (rs = 0.544; *p* < 0.001); MMSE and VFT (rs = 0.361; *p* = 0.014); PFEFFER and GDS (rs = 0.356; *p* = 0.015). There was also a negative correlation between CDT and age (rs = −0.338; *p* = 0.021); VFT and age (rs = −0.313; *p* = 0.034); CDT and PFEFFER (rs = −0.300; *p* = 0.043); MMSE and PFEFFER (rs = −0.487; *p* = 0.001); VFT and PFEFFER (rs = −0.599; *p* < 0.001).

**Table 4:** Spearman correlation among the variables of interest.

		AGE	GDS-15	GAI	IVCF-20	CDT	MMSE	VFT	PFEFFER	KATZ
AGE	rs	1								
	*p*-value									
GDS-15	rs	−0.279	1							
	*p*-value	0.061								
GAI	rs	−0.236	−0.031	1						
	*p*-value	0.115	0.838							
IVCF-20	rs	0.246	0.344^*^	0.243	1					
	*p*-value	0.100	0.019	0.103						
CDT	rs	−0.338^*^	0.087	−0.287	−0.473^*^	1				
	*p*-value	0.021	0.566	0.053	0.001					
MMSE	rs	−0.091	−0.137	−0.258	−0.581^*^	0.544^*^	1			
	*p*-value	0.547	0.362	0.083	<0.001	<0.001				
VFT	rs	−0.313^*^	−0.074	−0.002	−0.601^*^	0.245	0.361^*^	1		
	*p*-value	0.034	0.626	0.987	<0.001	0.101	0.014			
PFEFFER	rs	0.063	0.356^*^	0.167	0.692^*^	−0.300^*^	−0.487^*^	−0.599^*^	1	
	*p*-value	0.677	0.015	0.268	<0.001	0.043	0.001	<0.001		
KATZ	rs	0.037	−0.137	−0.137	−0.020	0.026	0.185	0.050	−0.177	1
	*p*-value	0.809	0.365	0.365	0.895	0.862	0.219	0.741	0.238	

Spearman’s correlation was applied to assess the correlation between variables of interest.

* *p*-value < 0.05 was considered significant.

## 4. Discussion

### 4.1. Main findings

In summary, our data support that the associations between frailty, cognitive impairment and functional disability were significant. In line with previous studies, we found a high frailty prevalence (41.31%) among older adults with affective disorders. Similarly, a systematic review showed that the prevalence of frailty in community-dwelling older adults with depression was 40.4% (22). In the outpatient context, the prevalence of frailty between depressed older adults was 37.7% and 37.5%, respectively (23, 24).

According to the literature, the link between physical frailty and cognitive impairment is so clear that the International Academy on Nutrition and Aging (I.A.N.A) and the International Association of Gerontology and Geriatrics (I.A.G.G) international consensus group introduced the concept of “cognitive frailty” to describe a diverse clinical condition characterized by the simultaneous presence of physical frailty and cognitive impairment excluding dementia (25). A meta-analysis, conducted by Zou (26), examined the correlation between cognitive frailty (*CF*) and depression among the elderly population and their findings indicated that around 46% of older adults with *CF* also experienced depression. Furthermore, they observed that *CF* in older adults is linked to a significantly increased risk for depression (twice the likelihood compared to individuals without *CF*). In the study of Feng (27), cognitively impaired frail individuals exhibited a notably higher prevalence and incidence of functional disability (12- to 13-fold increase). A systematic review conducted a comprehensive analysis of the existing literature and determined that the pooled prevalence of cognitive frailty in older adults residing in community settings was 9% and through stratified analysis, it was observed that the prevalence of cognitive frailty was higher among older women (28).

Based on the preliminary findings, this study found that multimorbidity, polypharmacy and the need for a caregiver increase the risk of frailty. Our data suggest that frailty prevalence among older adults with multimorbidity could be 5 times higher than among those without multimorbidity. Previous studies in the literature have shown that multimorbidity and polypharmacy are two related conditions in frail older adults (29, 30). In addition, this study found that being married could be a protection factor for frailty as cited previously in a review study (31).

### 4.2. Strengths and limitations

Some of the strengths of this paper are that our data are in line to previous studies reported in the literature that frailty is prevalent in older adults with affective disorders (4, 6) and is correlated to cognitive impairment and disability for IADLs (11, 32). This study found that there was a negative correlation between frailty and cognitive impairment (CDT; MMSE; and VFT), which means that the lower the scores on cognitive assessment, the higher the probability of frailty. There was no statistically significant correlation between anxiety symptoms (GAI) and cognitive or functional decline in BADLs (KATZ). On the other hand, previous studies have already showed that anxiety interferes in cognitive functioning and could be a predictor for frailty (6, 10). This finding may have occurred due to the small number of participants in the study (*n* = 46). However, in order to minimize a potential sampling bias, we intend to follow up the patients/participants until the total sample have been completed (*n* = 153).

First, we have chosen the IVCF-20 based on the judgment of being the most appropriate to our “real-world” research and clinical practice. Second, the IVCF-20 has already been validated for the use in our country (14) with good clinimetric properties as cited previously (13). Third, we decided to assess frailty using the IVCF-20 based on the issues related to the validation for our location and the suitability for our context. However, some limitations about frailty assessment using the IVCF-20 need to be acknowledged. Some domains assessed by IVCF-20 could lead to an overlap of measurements to evaluate affective disorders (GDS-15 and GAI); cognitive impairment (MMSE; CDT; VFT); disability for IADLs (PFEFFER) and for BALDs (KATZ). It is important to emphasize that the 2 questions used by IVCF-20 for screening “mood” are quite different and do not fulfill all criteria used for the screening of geriatric depression or anxiety. On the other hand, in order to evaluate affective disorders in our sample, we used GDS-15 and GAI, respectively. In addition, 3 questions used by IVCF-20 to assess “cognition” are more related to a subjective cognitive decline (SCD) than cognitive impairment evaluated objectively by a known brief cognitive screening. A recent systematic review found that in terms of cognitive assessment tools, 21 of them showed a higher accuracy for the detection of suspected cognitive decline in older adults with low levels of formal education, including MMSE, VFT, CDT tools that were applied in our sample (33).

In line with these issues, the functional assessment (secondary outcome) using Pfeffer Questionnaire or Functional Assessment Questionnaire (FAQ) and Katz Index are instruments that evaluate separately disability for IADLs (10 items) and BALDs (5 items), respectively. Comparing with the only 2 items considered by IVCF-15 to assess “functional disabilities” these tools are more specific and valid for the use in clinical practice and future research. Finally, IVCF-20 analyze a domain called “morbidities” including 3 items together (“Five or more chronic diseases” or “daily use of five or more different drugs” or “hospitalization in the last six months”) we analyzed these clinical variables (“multimorbidity”; “polypharmacy”; and “hospitalization during past 6 months”), separately.

### 4.3. Clinical implications

In conclusion, we decided to apply these validated instruments in order to investigate the associations of frailty with different domains: mood, cognition, disability, and clinical multimorbidity. Therefore, the early detection and control of these conditions could reverse or reduce the impact of frailty on cognitive impairment and functional disability, promoting a better quality of life in the context of healthy aging. Assessing frailty in a multidimensional context is essential and using a rapid assessment frailty tool such as IVCF-20 can provide easy and feasible evaluation in clinical practice.

## Data availability statement

The raw data supporting the conclusions of this article will be made available by the authors, without undue reservation.

## Ethics statement

The studies involving human participants were reviewed and approved by Complexo Hospital de Clínicas, Universidade Federal do Paraná (CHC-UFPR). The patients/participants provided their written informed consent to participate in this study.

## Author contributions

AM and MB have made substantial contributions to the conception and design of the work, have participated in the acquisition, analysis, and interpretation of the data, and have drafted the manuscript. All authors contributed to the article and approved the submitted version.

## Conflict of interest

The authors declare that the research was conducted in the absence of any commercial or financial relationships that could be construed as a potential conflict of interest.

## Publisher’s note

All claims expressed in this article are solely those of the authors and do not necessarily represent those of their affiliated organizations, or those of the publisher, the editors and the reviewers. Any product that may be evaluated in this article, or claim that may be made by its manufacturer, is not guaranteed or endorsed by the publisher.
